# Correlation of duration of β-1,3/1,6-glucan administration with the effectiveness of immune stimulation in juvenile vimba bream *(Vimba vimba)*

**DOI:** 10.2478/jvetres-2026-0023

**Published:** 2026-04-18

**Authors:** Barbara Kazuń, Krzysztof Kazuń, Joanna Małaczewska, Rafał Kaminski, Justyna Sikorska, Jacek Wolnicki

**Affiliations:** 1 Department of Ichthyopathology and Fish Health Protection, Pond Fishery Department, National Inland Fisheries Research Institute, Żabieniec, 05-500 Piaseczno, Poland; 2 Department of Microbiology and Clinical Immunology, Faculty of Veterinary Medicine, University of Warmia and Mazury in Olsztyn, 10-719 Olsztyn, Poland; 3 Pond Fishery Department, National Inland Fisheries Research Institute, Żabieniec, 05-500 Piaseczno, Poland

**Keywords:** aquaculture, β-glucan, fish, innate immunity, time-dependent effects

## Abstract

**Introduction:**

The purpose of this study was to determine how the duration of administration of β-glucans derived from the yeast *Saccharomyces cerevisiae* modifies cellular immunity in one-year-old leuciscids, specifically vimba bream (*Vimba vimba*).

**Material and Methods:**

The effects of two treatments were compared at three endpoints using a 2 × 3 factorial design, all groups in triplicate aquaria (n = 3). Kept in controlled recirculating aquaculture system conditions, three experimental groups were fed for two, four or eight weeks a commercial starter feed, and three other groups received the same feed supplemented with a commercial formulation of β-glucan polysaccharides from the cell wall of *Saccharomyces cerevisiae* yeast at 0.02%. Results for fish taking β-glucan were compared to the respective control groups of fish not given it.

**Results:**

Two-week administration of β-glucan in the diet resulted in significantly higher pinocytosis, respiratory burst activity and potential killing activity of head-kidney phagocytes in supplemented fish compared to phagocytic activities in fish not receiving β-glucan. However, in the groups receiving β-glucan for four and eight weeks, this activity was significantly lower than in the respective control groups. After eight weeks, T-lymphocyte activity was also lower.

**Conclusion:**

Under controlled recirculating aquaculture system conditions favourable for fish growth, the most beneficial effect of dietary β-glucan supplementation on vimba bream immune cells appears after two weeks. Extension of this period to four or eight weeks loses beneficial effects, and even significantly decreases some immune parameters. This finding is critical for animal nutrition, where glucans or their sources are increasingly used as permanent components of feed.

## Introduction

Immunomodulators are widely used in aquaculture as supplements for their beneficial effects on fish health and concomitant safety of use. They are a diverse group of substances that include enzymes, antioxidants, probiotics, prebiotics, synbiotics, dyes and attractants (27). Some immunomodulators benefit productivity by improving feed utilisation and aiding digestive processes and nutrient absorption. All of them stimulate the immune system and thus contribute to the prevention of infectious diseases. Sometimes immunomodulators are used in aquaculture as a permanent component of fish feed. The most popular among them are β-glucans, polysaccharides derived from the cell wall of the yeast *Saccharomyces cerevisiae*. Their ability to induce an immune system response and increase anti-infective immunity in many fish species has been confirmed in the literature. The use of β-glucans also increases the stress tolerance of animals (4, 15).

Cypriniform fishes (Cypriniformes) are the most abundant group of freshwater fishes in the world and are characterised by high species diversity. This group includes a leuciscid (Leuciscidae) rheophilic species, the vimba bream (*Vimba vimba*) (21). Until the 1950s, vimba bream had high economic value in Poland. According to the International Union for Conservation of Nature, the vimba bream has been classified as a least concern (LC) species in Europe. In Poland, however, it is recognised as a critically endangered species, and therefore, many actions are being taken for its restoration in areas of its original occurrence. One of them is the stocking of rivers with fish reared under controlled conditions to compensate for the shortfall in recruitment from natural spawning. However, fish originating from controlled conditions have very limited contact with only a few pathogens present in the environment. Therefore, they are unable to develop effective immune system mechanisms to protect them from the pathogens they will inevitably encounter after stocking in natural conditions. The use of immunostimulants might prepare them for these challenges.

In a previous study, long-term (55-day) administration of β-glucans derived from *S. cerevisiae* to juvenile vimba bream had an adverse effect on some immune parameters (8). The aim of the present work was to determine how the duration of β-glucan administration modifies cellular immunity in juveniles of this fish species.

## Material and Methods

### Experimental fish

The pooled progeny of three female and five male vimba bream were used in the experiment. Both spawners and their offspring were raised under controlled conditions in the laboratories of the National Inland Fisheries Research Institute located in Żabieniec, Poland. The experiment was carried out in the same location. It began when the experimental fish were one year old and their mean (± SD) body weight (BW), total length (TL) and condition factor K were 1.18 ± 0.22 g, 56.1 ± 3.5 mm and 0.66 ± 0.04, respectively.

### Experimental design

The effects of two feeding treatments on fish growth, feed utilisation and immunological parameters were assessed at three endpoints in a 2 × 3 factorial design; thus, six independent groups of fish were formed. Each group was kept in three replicate aquaria. The experimental fish were fed commercial starter feed (Aller Futura EX GR 0.5–1.0 mm, 60.0% crude protein, 15.0% total lipids and 21.2 MJ/kg gross energy; Aller Aqua, Christiansfeld, Denmark; groups C2, C4 and C8) or the same feed supplemented with 0.02% Leiber Beta-S (high-purity 1.3–1.6-beta-D-glucan molecules from the cell wall of *S. cerevisiae*, Leiber, Bramsche, Germany; groups G2, G4 and G8), as described by Kazuń *et al*. (8). The fish were fed their respective diets for 2, 4 or 8 weeks.

### Experimental conditions

The fish were stocked into 18 flow-through glass aquaria (V = 20 L) at 30 fish per aquarium. Prior to stocking, the fish were checked for the absence of body deformities and selected according to their BW to unify their initial size distribution in all the experimental groups (18).

All the aquaria were continuously supplied with filtered, heated and aerated water from a recirculating aquaculture system at a flow rate of approximately 0.25 L/min. The water temperature and dissolved oxygen concentration inside the aquaria were measured twice a day. The water temperature was 25.0°C (range 24.5–25.5°C). The water in the aquaria was continuously aerated with air stones to maintain the oxygen saturation in the range of 70–95%. Other water quality parameters were monitored weekly. The concentrations of total ammonia and nitrites were less than 0.2 mg/L and 0.04 mg/L, respectively. The water conductivity ranged 517–536 μS/cm, and the pH ranged 7.92–8.41. Aquaria were illuminated from 08:00 to 21:00 by fluorescent tube lights at approximately 600 lx at the water surface.

Equal portions of feed were provided to the fish manually five times a day every 3 h between 08:00 and 20:00. The daily food ratio per aquarium was initially 1.3 g, *i.e*. 3.7% of the fish biomass (FB). During the experiment it was increased twice to keep pace with fish growth: on the 15^th^ day to 1.88 g (3.4% FB) and on the 29^th^ day to 3.0 g (3.4% FB).

### Sample collection

At the end of the experiment, all the fish were anaesthetised, and their individual BW and TL were measured. All 30 fish from each experimental aquarium were euthanised by immersion in an overdose (150 mg/L) of unbuffered tricaine methanesulphonate solution (MS-222; Sigma-Aldrich, St. Louis, MO, USA), and fish head kidney samples were taken.

### Isolation of immune cells

The samples of head kidneys taken from all vimba bream individuals from each experimental aquarium were pooled because individual fish of relatively small size provide too little biological material. Immune cells were isolated using density gradient centrifugation (Histopaque 1077) and cultured in RPMI-1640 medium supplemented with 10% foetal calf serum and 1% antibiotic-antimycotic solution (all reagents from Sigma-Aldrich) at 22°C (11). Macrophage activity was evaluated using a pinocytosis assay and respiratory burst activity (RBA) and potential killing activity (PKA) tests, while the 3-(4,5-dimethylthiazol-2-yl)-2,5-diphenyltetrazolium bromide (MTT) reduction assay was used to assess the proliferative activity of lymphocytes. All the samples were analysed in triplicate.

### Pinocytosis assay – neutral red uptake

The pinocytosis assay was performed using a commercial TOX-4 kit (Sigma-Aldrich), as described previously (11). Briefly, following overnight incubation at 22°C, the adherent cells were incubated for 60 min in fresh medium containing 0.033% neutral red dye. Then, the cells were washed with PBS, and the pinocytosed dye was extracted from the cells using a 1% acetic acid solution in 50% ethanol. The OD of the samples was measured at a wavelength of 540 nm, with 690 nm as the reference wavelength. The pinocytosis of group G cells was expressed as a percentage of the group C cell activity.

### Respiratory burst activity and potential killing activity tests

The adherent head-kidney cells were stimulated after overnight incubation at 22°C with phorbol myristate acetate (PMA, Sigma–Aldrich, 1 μg/mL) or live *Aeromonas hydrophila* (1×10^8^, cells/mL) for 60 min in medium containing 0.1% nitro blue tetrazolium (NBT, Sigma-Aldrich) at 22°C (11). Yellow NBT dye was reduced to blue formazan by activated phagocytes. Then, the OD of the samples was measured colourimetrically at 620 nm. The results were expressed as the stimulation index (SI), which was calculated by dividing the mean OD of PMA-(RBA test) or bacteria-stimulated cells (PKA test) by the OD of the unstimulated control cells.

### Proliferative response of lymphocytes – MTT reduction assay

The mitogenic response of vimba lymphocytes was determined using the MTT colorimetric assay, as described by Kazuń *et al*. (11). Head-kidney cells were cultured for 72 h at 22°C in medium supplemented with concanavalin A (ConA) as a T-cell mitogen or lipopolysaccharide from *Escherichia coli* as a B-cell mitogen. After incubation, MTT solution was added to each well, and the plate was incubated for the next 3 h (all reagents from Sigma-Aldrich). Then, the OD was measured at a wavelength of 570 nm, with 640 nm as the reference wavelength. The results are expressed as the SI, which was calculated by dividing the mean OD of the mitogen-stimulated cells by the OD of the unstimulated control cells.

## Statistical analysis

The difference between the means was tested at the 5% probability level using the unpaired Student’s *t*-test. All the statistical analyses were carried out using the GraphPad Prism 7 software package (GraphPad Software, San Diego, CA, USA).

## Results

### Evaluation of immune cell activity

**Fig. 1.**
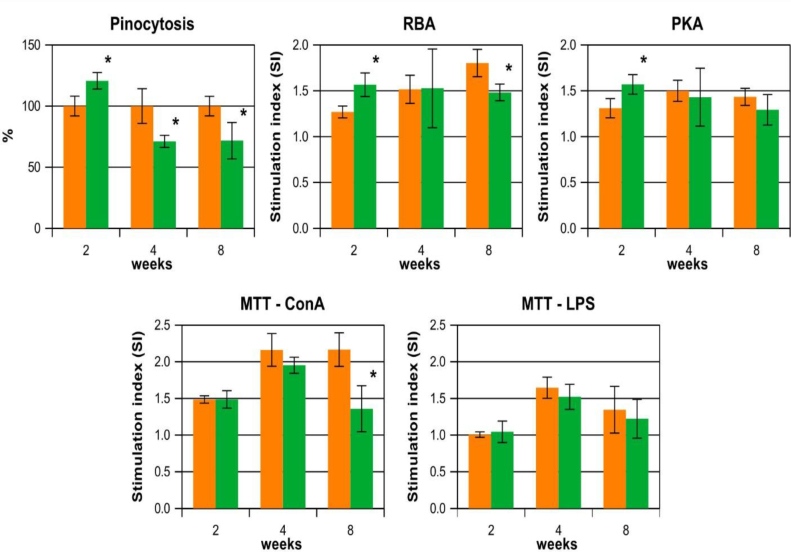
Comparison of means (whiskers show SD, n = 3) of parameters of head-kidney immune cell activity in juvenile vimba bream fed commercial dry feed (diet C, orange bars) or the same feed supplemented with 0.02% β-1,3/1,6-glucan (diet G, green bars) for 2, 4 or 8 weeks. RBA – respiratory burst activity; PKA – potential killing activity; MTT – 3-(4,5-dimethylthiazol-2-yl)-2,5-diphenyltetrazolium bromide; LPS – lipopolysaccharide; * – significant difference between a pair of means (unpaired Student’s *t* test, P-value < 0.05)

Two weeks of providing the fish with feed supplemented with β-glucans resulted in increased pinocytosis, RBA and PKA in group G2 as compared to group C2 (Fig. 1).

After four weeks of β-glucan administration, RBA and PKA levels in group G4 were similar to those in group C4, while the mean level of pinocytosis was significantly lower. After eight weeks of treatment, pinocytosis, RBA and T-lymphocyte proliferative activity (ConA) were significantly lower in group G8 than in group C8 (Fig. 1).

### Fish growth and feed utilisation

All fish survived the experiment. There was no significant difference (P-value > 0.05) between groups fed different diets for the same time for the fish condition factor, growth parameters or feed conversion ratio (Table 1).

**Table 1.** Condition, growth and feed conversion in juvenile vimba bream fed for 2, 4 or 8 weeks with dry feed (C) or the same feed with 0.02% β-1,3/1,6-glucan (G)

Parameter	Group		P-value
	C2	G2	
Condition factor K	0.743 ± 0.010	0.740 ± 0.001	0.560
Relative growth rate, %/d	3.231 ± 0.118	3.258 ± 0.062	0.743
Daily increment in total length, mm/d	0.481 ± 0.048	0.477 ± 0.008	0.898
Feed conversion ratio	0.918 ± 0.037	0.891 ± 0.014	0.304
	C4	G4	
Condition factor K	0.788 ± 0.015	0.791 ± 0.005	0.751
Relative growth rate, %/d	3.327 ± 0.024	3.271 ± 0.080	0.314
Daily increment in total length, mm/d	0.574 ± 0.004	0.560 ± 0.024	0.380
Feed conversion ratio	0.869 ± 0.015	0.899 ± 0.038	0.280
	C8	G8	
Condition factor K	0.818 ± 0.004	0.811 ± 0.010	0.322
Relative growth rate, %/d	3.045 ± 0.024	3.016 ± 0.031	0.278
Daily increment in total length, mm/d	0.624 ± 0.006	0.624 ± 0.013	0.984
Feed conversion ratio	0.934 ± 0.009	0.944 ± 0.027	0.579

1 The data are presented as means ± SD, n = 3, unpaired Student’s *t*-test. C – fish given Aller Futura EX GR 0.5–1.0 mm granule-size commercial dry feed; G – fish given the commercial dry feed supplemented with 0.02% Leiber Beta-S β-1,3/1,6 glucan. The number following the letter specifying the fish diet indicates the duration of treatment in weeks

## Discussion

Numerous studies on the use of β-glucans in aquaculture have primarily focused on demonstrating their immunostimulatory effects, with the aim of using them to boost animals’ resistance to infectious diseases. However, there are few scientific reports detailing the impact of different feeding regimes involving β-glucan-enriched feeds on the effectiveness of immunostimulation.

The results obtained in the present study indicate that the most beneficial effect on the activity of fish immune cells was obtained with the use of a commercial diet supplemented with β-glucans for two weeks. A considerable prolongation of this period seemed to result in a reduction in immune parameters in fish stimulated with β-glucans relative to those in the control group, indicating immunosuppression. This was particularly evident in the activity of phagocytic cells, which are the main target of β-glucans. As part of the body’s innate immune system, these cells are the first line of defence at an early stage of infection, and their ability to phagocytose and kill microorganisms often stops pathogens at the site of infection, preventing the disease from progressing. In addition, phagocytic cells are also involved in cytokine production and antigen presentation to T lymphocytes, which is associated with the activation of the acquired immune response (17).

There are many reports on the beneficial effects of short-term diet supplementation with β-glucans on innate immune parameters in various fish species (9, 10, 15, 23, 24). In contrast, the results of studies on long-term supplementation are sometimes divergent. For example, Koch *et al*. (12) reported that the administration of glucans to Nile tilapia (*Oreochromis niloticus*) positively influenced the innate immune response and disease resistance, regardless of the period of administration (15 to 45 days). An increase in RBA was also reported by Gopalakannan and Arul (5) and Lin *et al*. (14) in common carp (*Cyprinus carpio*) after dietary supplementation with β-glucans for 60 and 56 days, respectively. However, several researchers have observed that prolonged β-glucan supplementation can lead to a gradual decrease in immune cell activity. In a study by Yoshida *et al*. (28), the greatest beneficial effect of continuous oral administration of β-glucan to North African catfish (*Clarias gariepinus*) on NBT-positive cell levels was observed after 30 days of supplementation. After an additional 15 days of supplementation, cell activity decreased from that of the control group to a statistically nonsignificant extent. Additionally, the results of different studies carried out on cyprinid fish, such as common carp, roho labeo (*Labeo rohita*) and koi carp (5, 14, 16), confirmed a gradual decrease in immune parameters after long-term continuous oral administration of β-glucans. Long-term administration of these substances may cause overstimulation of immune cells, leading to their exhaustion and subsequent immunosuppression by negative feedback regulation (4). However, in our experiment, the eight-week duration feeding fish with β-glucan-supplemented feed negatively affected not only the activity of phagocytes, which are associated with innate immunity, but also that of T lymphocytes, which are involved in adaptive immunity. This finding is crucial in animal nutrition, where glucans or a source of glucans are increasingly used as a permanent component of feed.

The activation of the immune system requires the allocation of some metabolic effort and may adversely affect the growth rate of fish (25). This may explain why, in our experiment, feed supplementation with β-glucans had no significant effect on the condition factor, growth rate or feed conversion ratio of the fish, regardless of the period of administration. Similar results were also shown in European seabass (*Dicentrarchus labrax*) (3) and tropical gar (*Atractosteus tropicus*) (20). However, a surprisingly large number of scientific reports show that the addition of β-glucans to feed can also have a beneficial effect on the growth rate of fish of different species (1, 2, 7, 13, 14, 19, 22, 26). Thus, it is still valid to conclude that the effect of β-glucan on the growth of fish is not fully understood, and possible factors responsible for this effect include the concentration of β-glucan in the diet and species-specific responses (6).

## Conclusion

The results of our study confirm that two-week administration of β-glucans in a dry diet to vimba bream has an immunostimulatory effect, but extending the treatment to four weeks or longer may have the opposite effect. This is important in animal nutrition, where glucans are increasingly used as a permanent component of feed. The timing of β-glucan administration should be considered when planning feeding protocols for fish.
